# Subspecies Classification and Comparative Genomic Analysis of *Lactobacillus kefiranofaciens* HL1 and M1 for Potential Niche-Specific Genes and Pathways

**DOI:** 10.3390/microorganisms10081637

**Published:** 2022-08-12

**Authors:** Sheng-Yao Wang, Yen-Po Chen, Ren-Feng Huang, Yi-Lu Wu, Shang-Tse Ho, Kuan-Yi Li, Koichi Watanabe, Ming-Ju Chen

**Affiliations:** 1 Department of Animal Science and Technology, National Taiwan University, Taipei 106037, Taiwan; 2 Department of Animal Science, The iEGG and Animal Biotechnology Center, National Chung Hsing University, Taichung 402204, Taiwan; 3 Department of Wood Based Materials and Design, National Chiayi University, Chiayi 600355, Taiwan

**Keywords:** *L. kefiranofaciens* subsp. *kefirgranum*, *L. kefiranofaciens* subsp. *kefiranofaciens*, subspecies classification, comparative genomic analysis

## Abstract

(1) Background: Strains HL1 and M1, isolated from kefir grains, have been tentatively identified, based on their partial 16S rRNA gene sequences, as *Lactobacillus kefiranofaciens*. The two strains demonstrated different health benefits. Therefore, not only the genetic factors exerting diverse functionalities in different *L. kefiranofaciens* strains, but also the potential niche-specific genes and pathways among the *L. kefiranofaciens* strains, should be identified. (2) Methods: Phenotypic and genotypic approaches were employed to identify strains HL1 and M1 at the subspecies level. For the further characterization of the probiotic properties of both strains, comparative genomic analyses were used. (3) Results: Both strains were identified as *L. kefiranofaciens* subsp. *kefirgranum*. According to the COG function category, dTDP-rhamnose and rhamnose-containing glycans were specifically detected in the *L. kefiranofaciens* subsp. *Kefirgranum* genomes. Three unique genes (*epsI*, *epsJ*, and *epsK*) encoding glycosyltransferase in the EPS gene cluster, and the ImpB/MucB/SamB family protein encoding gene were found in HL1 and M1. The specific ability to degrade arginine via the ADI pathway was found in HL1. The presence of the complete glycogen metabolism (*glg*) operon in the *L. kefiranofaciens* strains suggested the importance of glycogen synthesis to enable colonization in kefir grains and extend survival under environmental stresses. (4) Conclusions: The obtained novel information on the potential genes and pathways for polysaccharide synthesis and other functionalities in our HL1 and M1 strains could be applied for further functionality predictions for potential probiotic screening.

## 1. Introduction

*Lactobacillus kefiranofaciens* was first described in 1988 by Fujisawa et al. [1] for homofermentative lactobacilli strains isolated from kefir grains. This species has been reported as a kefiran (exopolysaccharide, EPS) producer in kefir grains. Kefiran can be used as a food grade additive to obtain fermented products due to its rheological properties, which enhance the apparent viscosity, storage and loss modulus of chemically acidified skim milk gels [2]. This phenomenon was strengthened by the heat treatment usually applied in the manufacturing of yogurts [3].

In 1994, *Lactobacillus kefirgranum* has published validly as a new species among the homofermentative lactobacilli strains from kefir grains [4]. However, Vacanneyt et al. (2004) reclassified *L. kefirgranum* as *L. kefiranofaciens* subsp. *kefirgranum*, since *L. kefirgranum* and *L. kefiranofaciens* show 100% 16S rRNA gene sequence similarity, DNA–DNA hybridization values of >79% and DNA G+C contents of 37–38 mol%, demonstrating that both species belonged to one species. Two subspecies, i.e., *L. kefiranofaciens* subsp. *kefiranofaciens* and *L. kefiranofaciens* subsp. *Kefirgranum*, could be differentiated with each other on the basis of the differences in their phenotypic features and the sodium dodecyl sulfate polyacrylamide gel electrophoresis (SDS-PAGE) profiles of whole-cell proteins [5]. *L. kefiranofaciens* subsp. *kefiranofaciens* presents transparent, glossy, convex and extremely slimy colonies on MLR medium under anaerobic condition with negative hydrolysis of aesculin, producing large amounts of polysaccharides. In contrast, *L. kefiranofaciens* subsp. *kefirgranum* possesses white, dry, compact, dull and bulging colonies with positive hydrolysis of aesculin. Flocculus or powdery sediment is observed in broth [1,4,5]. However, to date, the genes and pathways involved in EPS production and other functions of *L. kefiranofaciens* between the two subspecies have not been comprehensively studied.

Previously, we isolated two strains, M1 and HL1, from kefir grains of different sources, and identified tentatively *L. kefiranofaciens* by polymerase chain reaction-denaturing gradient gel electrophoresis (PCR-DGGE) targeting the V3 variable region of the 16S rRNA gene [6]. The *L. kefiranofaciens* M1 strain has been demonstrated to have immune-modulating activity in vitro [7], anti-allergic [8], and anti-asthma [9] properties in a murine model regarding Th1/Th2 balance, to enhance regulatory T cells (Treg) and upregulating genes involved in immune responses, inflammation and cell adhesion, and to decrease the expression of genes associated with the classic complement and lectin-induced pathways. For intestinal barrier protection and anti-colitis effects, *L. kefiranofaciens* M1 improved epithelial barrier function in vitro by increasing the transepithelial electrical resistance (TEER) and significantly upregulating the level of the chemokine ligand CCL-20 [10]. Additionally, the administration of *L. kefiranofaciens* M1 with a high fat diet has obesity effects affecting adipogenesis, lipogenesis and inflammation by regulating the expression of metabolites [11,12,13]. The *L. kefiranofaciens* HL1 strain exhibited anti-oxidative and anti-aging properties by modulating short-chain fatty acids (SCFA), which may regulate antioxidant enzymes by inducing the expression of nuclear factor erythroid 2-related factor 2 (Nrf2)/heme oxygenase-1 (HO-1), inhibiting cell apoptosis and causing brain injury [14]. Additionally, *L. kefiranofaciens* HL1 also improved muscle strength and mass by regulating blood glucose, lactate and catalase activity in mice (unpublished data). Therefore, the genetic factors exerting diverse functionalities in different *L. kefiranofaciens* strains should be identified.

Thus, in the present study, we aimed to: (i) classify our *L. kefiranofaciens* strains, i.e., HL1 and M1, at the subspecies level; and (ii) characterize both strains by whole genome sequence analyses in order to elucidate their probiotic properties. The results obtained from this study not only enable the assessment of the probiotic potential of *L. kefiranofaciens* strains HL1 and M1, but also provide a fundamental understanding of the specific features of the two subspecies and strains of *L. kefiranofaciens* for further applications.

## 2. Materials and Methods

### 2.1. L. kefiranofaciens Strains and Culture Conditions

*L. kefiranofaciens* strains M1 and HL1 were previously isolated from kefir grains [6]. *L. kefiranofaciens* subsp. *kefiranofaciens* BCRC 16059^T^ (=ATCC 43781^T^) and *L. kefiranofaciens* subsp. *kefirgranum* BCRC 80410^T^ (=DSM 10550^T^) were obtained from the Bioresource Collection and Research Center (BCRC, Food Industry Research and Development Institute, Hsinchu, Taiwan). Four *L. kefiranofaciens* strains were cultured in de Man, Rogosa, and Sharp broth (MRS broth, Acumedia Manufacture, Lansing, MI, USA) and incubated at 30 °C for 36 h.

### 2.2. Subspecies Identification

#### 2.2.1. Phenotypic Characterization

Cell morphology was observed by microscopy after growth in MRS broth at 30 °C for 36 h. Gram-staining was performed using a Gram-staining kit (Sigma-Aldrich, St. Louis, MO, USA). Colony morphology was observed by steromicroscopy after growth on MRS agar at 30 °C under anaerobic conditions for 72 h. Carbohydrate fermentation was determined using API 50 CHL system (bioMérieux, Marcy-l’Etoile, France), according to the manufacturer’s instructions. The whole cell protein profile was analyzed as described previously [15], in three steps: cell protein extraction, protein quantification using a protein assay kit (Bio-Rad Protein Assay Kit, Bio-Rad, Hercules, CA, USA) and protein profiling using SDS-PAGE. The banding patterns were clustered together using the unweighted pair group method with arithmetic mean (UPGMA) algorithm. The evolutionary distances were computed using the *p*-distance method and are in the unit of the number of base differences per site.

#### 2.2.2. Genotypic Characterization

Genotypic characterization was performed by 16S rRNA and housekeeping gene sequence analysis, enterobacterial repetitive intergenic consensus polymerase chain reaction (ERIC-PCR) and randomly amplified polymorphic DNA (RAPD) fingerprinting [16], as well as by whole genome sequence-based methods, e.g., based on the average nucleotide identity (ANI) values, digital DNA–DNA hybridization (dDDH) and phylogenomic analysis.

##### DNA Extraction and 16S rRNA and Housekeeping Gene Sequence Analysis

The genomic DNA of four *L. kefiranofaciens* strains were extracted using the Genomic DNA Mini Kit (Geneais Biotech, Taipei, Taiwan). The 16S rRNA gene was amplified with the 8F and 15R primers [15]. Full-length sequencing of the 16S rRNA gene was conducted with the 350F, 520R and 930F primers [16] (Table S1). The amplification and sequencing of two housekeeping genes, i.e., the phenylalanyl-tRNA synthase alpha (*phe*S) and RNA polymerase alpha subunits (*rpo*A), were conducted as described by Naser et al. [17,18]. Briefly, two pairs of primers, pheS 21F/pheS 23R and rpoA 21F/rpoA 23R, were applied (Table S1). Consensus sequences were then determined. After sequencing (Genomics BioSci & Tech Co., Ltd., New Taipei, Taiwan), the data were assembled using Chromas v2.23 (Technelysium Pty. Ltd., Brisbane, Australia). A phylogenetic tree was constructed by the neighbor-joining method [19] with Kimura’s two-parameter model [20] using the MEGA7 v7.0.14 software [21]. The statistical reliability of the trees was evaluated by bootstrap analysis of 1000 replicates [22].

##### ERIC-PCR

The genomic DNA of four strains were amplified using the ERIC 1 and ERIC 2 pair of primers, as described previously [23] (Table S1). The PCR products were electrophoresed on 1.5% (wt/vol) agarose (Fisher Biotech, Fair Lawn, NJ, USA) gel electrophoresis (BioDoc-It R 220 Imaging System, UVP LLC., Upland, CA, USA) with ethidium bromide staining. The process was repeated twice to verify the accuracy of the results.

##### RAPD

The extracted genomic DNA was used as a template in subsequent PCR amplifications. Five primers [24], i.e., RAPD-A, RAPD-B, RAPD-E, RAPD-G, and RAPD-I, with arbitrary nucleotide sequences were used (Table S1). The RAPD products were electrophoresed on 1.5% (wt/vol) agarose gel. The process was performed twice. The banding patterns were clustered using the UPGMA algorithm with Dice coefficients using the Dolphin-1D software (Wealtec Corp., Sparks, NV, USA).

##### Phylogenomic Analyses

Phylogenomic trees based on the whole genome sequences were constructed using the Type (Strain) Genome Server (TYGS; https://tygs.dsmz.de/, accessed on 22 February 2022), and the core gene multilocus sequence typing (cgMLST) analysis of the 1674 core genes for the six *L. kefiranofaciens* strains was undertaken using the PGAdb-builder (http://wgmlstdb.inst.nsysu.edu.tw/index.php, accessed on 19 December 2021).

### 2.3. Whole Genome Sequencing, Assembly and Annotation

#### 2.3.1. Genome Sequencing and Assembly

The whole genomes of *L. kefiranofaciens* M1 and HL1 were sequenced with Nanopore (MinION, Oxford Nonopore Technologies, Oxford, UK) and Illumina MiSeq (Illumina, San Diego, CA, USA) (301 base, paired end reads). The Illumina raw data were trimmed to remove adapters, low quality sequences (Q20) and ambiguous bases. The nanopore reads were used to perform de novo assembly using NECAT program (https://github.com/xiaochunle/necat, accessed on 8 October 2021), and the contig with trimmed NovaSeq reads was corrected using CLC Genomics Workbench. Gap closing was performed using PCR and Sanger sequencing. The genome information of HL1 and M1 was deposited in the GenBank database under the accession nos. GCA_023674385.1 and GCA_023674405.1, respectively.

#### 2.3.2. Annotation and Comparative Analysis

Coding DNA sequences (CDSs), transfer RNAs (tRNAs), ribosomal RNAs (rRNAs) and transfer-messenger RNAs (tmRNAs) were predicted and annotated using the Prokka software v1.14.5 (https://vicbioinformatics.com/software.prokka.shtml, accessed on 20 October 2021), and with the NCBI databases (http://www.ncbi.nlm.nih.gov/, accessed on 20 October 2021). Circular maps of the assembled genomes were visualized using the DNAPlotter software (https://github.com/sanger-pathogens/Artemis, accessed on 28 October 2021). Functional annotation of CDSs was performed by Rapid Annotation using the Subsystem Technology (RAST) Prokaryotic Genome Annotation Server (http://rast.nmpdr.org/, accessed on 9 November 2021) with RASTtk annotation scheme and eggNOG-mapper (http://eggnog-mapper.embl.de/, accessed on 20 October 2021) and with the NCBI database. The Gene Ontology (GO) annotation of the Open Reading Frames (ORFs) was realized using Fast Annotation v1.2.2 (https://github.com/UoA-CARES/FastAnnotation/releases/tag/v1.2.2a, accessed on 2 December 2021), and the pathway mapping was performed on the KEGG Automatic Annotation Server (KAAS; https://www.genome.jp/kegg/kaas, accessed on 14 December 2021) [25,26,27]. For our comparative genomic analysis of HL1 and M1, the genome sequences of two *L. kefiranofaciens* subsp. *kefiranofaciens* strains (ATCC 43761^T^ (GCA_900103655.1) and ZW3 (GCA_000214785.1)), and two *L. kefiranofaciens* subsp. *kefirgranum* strains (DSM 10550^T^ (GCA_001434195.1) and KR (GCA_002276565.1)) were obtained from the NCBI database and used as references. The annotation files (GFF3) of HL1 and M1 and the reference strains were generated using the Prokka software and were employed for pan-genome analysis using Roary v3.11.2 (https://github.com/sanger-pathogens/Roary/, accessed on 21 December 2021). A Venn diagram of the unique/shared genetic content was generated with the InteractiVenn (http://www.interactivenn.net/, accessed on 20 October 2021). The MAUVE alignment tool (http://darlinglab.org/mauve/mouve.html, accessed on 5 December 2021) was used for multiple genome sequence alignment and visualization.

## 3. Results and Discussion

### 3.1. Subspecies Identification of HL1 and M1

For the classification of *L. kefiranofaciens* strains HL1 and M1 at the subspecies level, phenotypic and genotypic characterizations were conducted with two reference strains (*L. kefiranofaciens* subsp. *kefiranofaciens* BCRC 16059^T^ and *L. kefiranofaciens* subsp. *kefirgranum* BCRC 80410^T^).

#### 3.1.1. Phenotypic Characterization

**Figure 1:**
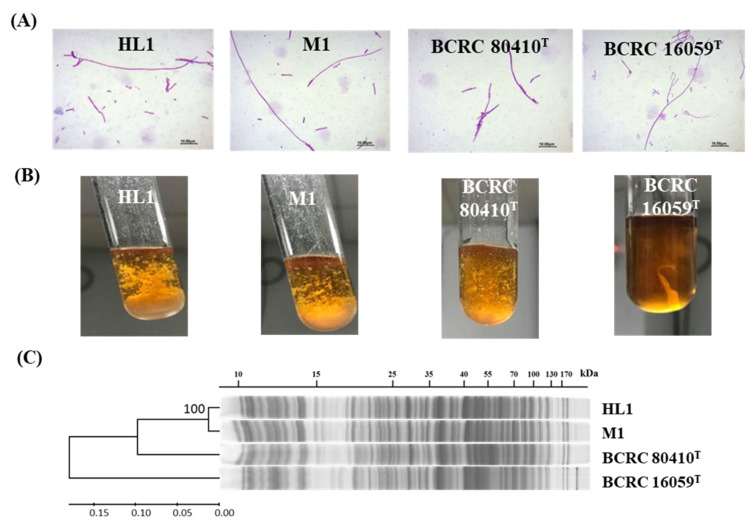
Phenotypic characteristics of *L. kefiranofaciens* strains HL1 and M1. (**A**) Morphology of cells grown in MRS broth; (**B**) Bacterial growth aspect in MRS broth; and (**C**) SDS-PAGE whole-cell protein profiles of four strains of *L. kefiranofaciens*. The banding patterns were clustered together using the UPGMA algorithm. The evolutionary distances are in the unit of the number of band differences per site, as calculated by p-distance.

First, we observed the cell morphology of *L. kefiranofaciens* HL1 and M1, as well as those of two reference strains, by microscopy. Cells of all four strains were Gram-positive rods ranging from 2 to 30 μm in length with no significant difference in morphology (Figure 1A). When cultured on MRL agar (replaced 1% glucose by 1% lactose), strains HL1, M1 and BCRC 80410^T^ demonstrated opaque and yellowish colonies with protrusions, whereas BCRC 16059^T^ showed a semi-transparent, white sticky surface (data not shown). In MRS broth, HL1, M1 and BCRC 80410^T^ showed powdery bacterial chunks with flocculation, while BCRC 16059^T^ showed a sticky appearance, indicative of high EPS production (Figure 1B). Our findings corresponded well with those of previous studies [28], i.e., that *L. kefiranofaciens* subsp. *kefirgranum* form dry, compact, dull bulging colonies, whereas *L. kefiranofaciens* subsp. *kefiranofaciens* have transparent, glossy, convex and extremely slimy colonies.

The carbohydrate fermentation characteristics of four strains, as determined using an API 50 CHL system, demonstrated diversity among strains in terms of the presence/contents of eleven carbohydrates (amygdalin, arbutin, d-cellobiose, gentibiose, d-maltose, d-melibiose, d-raffinose, salicin, d-sucrose, d-trehalose, and aesculin) (Table 1). All strains produced acid from d-fructose, d-galactose, d-glucose, d-lactose, d-mannose and *N*-acetylglucosamine, whereas none produced acid from the remaining 32 substrates according to the API 50 CHL system. Strains HL1, M1 and *L. kefiranofaciens* subsp. *kefirgranum* BCRC 80410^T^ hydrolyzed aesculin, whereas *L. kefiranofaciens* subsp. *kefiranofaciens* BCRC 16059^T^ did not. The result regarding aesculin hydrolysis was consistent with previous studies [1,4,28]. The fermentation patterns of carbohydrates suggested that *L. kefiranofaciens* strains HL1 and M1 may belong to the *kefirgranum* subspecies.

The SDS-PAGE whole cell protein profiles revealed that HL1 and M1 were closely related to each other in terms of the composition of their cell wall proteins. Additionally, strains HL1, M1 and *L. kefiranofaciens* subsp. *kefirgranum* BCRC 80410^T^ were bundled in a cluster and distinct from *L. kefiranofaciens* subsp. *kefiranofaciens* BCRC 16059^T^ on the basis of the three unique banding patterns in regions of 15–20, 30–35 and 170 kDa (Figure 1C and Supplementary Figure S1). This finding corresponded well to a previous study [5] which noted that SDS-PAGE profiles of whole-cell proteins could be used to differentiate the strains of *L. kefiranofaciens* at the subspecies level into two subspecies, i.e., *L. kefiranofaciens* subsp. *kefiranofaciens* and *L. kefiranofaciens* subsp. *kefirgranum*.

#### 3.1.2. Genotypic Characterization

**Figure 2:**
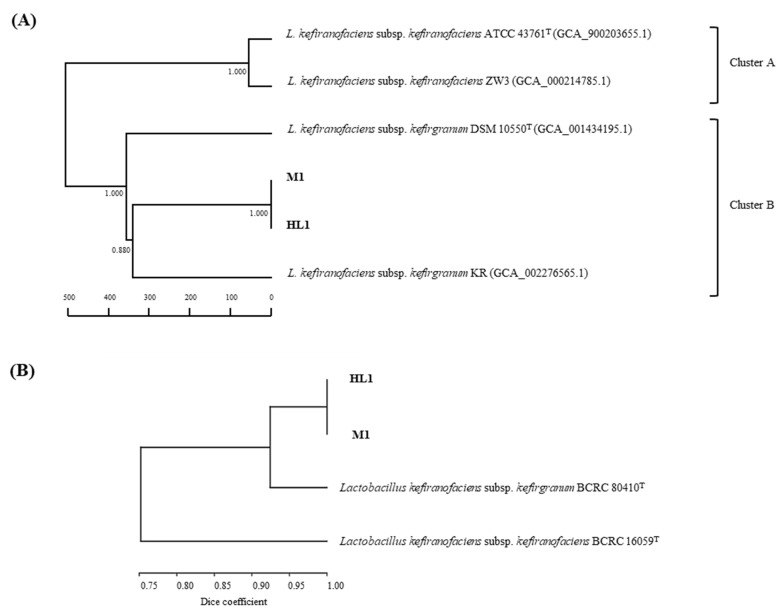
Genotypic characterization of *L. kefiranofaciens* HL1 and M1. (**A**) Phylogenomic tree of *L. kefiranofaciens* strains using the core gene multilocus sequence typing (cgMLST) of the 1674 core genes, applying the unweighted pair group method with arithmetic mean (UPGMA). Bar, allele numbers. (**B**) The enterobacterial repetitive intergenic consensus (ERIC)-PCR and five randomly amplified polymorphic DNA (RAPD) profiles of *L. kefiranofaciens* HL1 and M1, as well as reference strains for each strain, were concatenated. The banding patterns were clustered together using the unweighted pair group method with the arithmetic mean (UPGMA) algorithm, using Dice coefficients.

The ERIC-PCR and RAPD fingerprinting methods are considered convenient discriminatory tools for measuring biodiversity in the genomes of bacterial strains at the strain level. To investigate the taxonomic position of HL1 and M1, we carried out genotypic characterizations, including sequence analyses of 16S rRNA and two housekeeping genes (*pheS* and *rpoA*), ERIC-PCR and RAPD fingerprinting and phylogenomic and core genome multilocus sequence typing (cgMLST) analyses. The average nucleotide identity (ANI) values and the digital DNA–DNA hybridization (dDDH) values were also calculated. HL1 shared 100% 16S rRNA, *pheS* and *rpoA* gene sequence similarities with M1 and the type strains of *L. kefiranofaciens* subsp. *kefiranofaciens* and *L. kefiranofaciens* subsp. *kefirgranum*. Through phylogenetic analyses based on these three gene sequences together with the two types strains, HL1 and M1 were found to be located in an independent cluster among the species in the genus *Lactobacillus* (Supplementary Figures S2 and S3). The phylogenomic tree based on whole genome sequences showed that the six *L. keifanofaciens* strains were included in the same cluster (Supplementary Figure S4). We also identified the HL1 and M1 strains based on the overall genome related index (ORGI), e.g., the ANI and dDDH values. All strains of *L. kefiranofaciens* (HL1, M1, ATCC 43761^T^, DSM 10550^T^, ZW3 and KR) shared >99.2% ANI values and >93.7% dDDH values, indicating that these six strains represent the same species (see Supplementary Table S2). However, based on a core gene multilocus sequence typing (cgMLST) analysis of the 1674 core genes, the six *L. kefiranofaciens* strains could be clearly divided into two clusters: Cluster A (comprising two *L. kefiranofaciens* subsp. *kefiranofaciens* strains, ATCC 43761^T^ and ZW3), and Cluster B (comprising HL1 and M1, and two *L. kefiranofaciens* subsp. *kefirgranum* strains, DSM 10550^T^ and KR) (Figure 2A). For further subspecies identification of HL1 and M1, the ERIC-PCR and RAPD fingerprinting approaches were applied. Using dendrogram analysis based on the concatenated ERIC-PCR and five RAPD profiles, it was found that HL1 shares 100% similarity with M1, with these two strains forming a distinct cluster with BCRC 80410^T^, demonstrating that HL1 and M1 belong to *L. kefiranofaciens* subsp. *kefirgranum*. This result was consistent with the result obtained by SDS-PAGE protein profiling (Figure 2B). The results from a previous study using various phylogenetic and genotypic approaches, including 16S rRNA gene sequence analysis and DNA–DNA hybridizations, did not find discriminating power for subspecies identification of *L. kefiranofaciens.* However, we successfully differentiated the strains of *L. kefiranofaciens* subsp. *kefirgranum* from *L. kefiranofaciens* subsp. *kefiranofaciens* using SDS-PAGE whole-cell protein profiling and the RAPD typing method, as well as cgMLST analysis.

The findings from this study demonstrated that phenotypic- and genotypic-based strain identification methods were extremely effective for the classification of *L. kefiranofaciens* into two subspecies. Consequently, we confirmed that our strains, HL1 and M1, were indeed *L. kefiranofaciens* subsp. *kefirgranum*.

### 3.2. Comparative Genomic Analysis of HL1 and M1

#### 3.2.1. Genome Features

**Figure 3:**
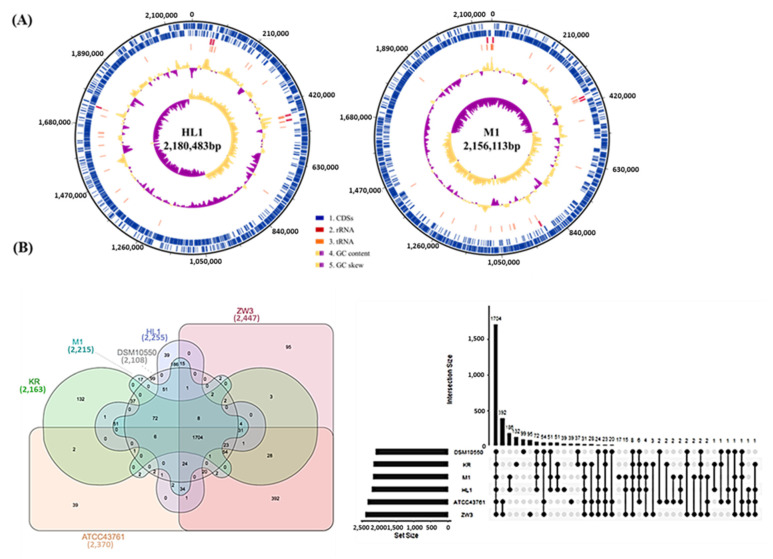
(**A**) Circular genome maps of *L. kefiranofaciens* HL1 and M1. The six circles (outer to inner) show the following. Circles 1 and 2 demonstrate the CDSs on the forward and reverse strands, respectively. Circles 3, 4, and 5 show the rRNAs and tRNAs and the GC content. Circle 6 represents the GC skew ((C − G)/(C + G)) curve (positive GC skew, orange; negative GC skew, violet); (**B**) Venn diagrams and Upset plot of coding sequences for the six *L. kefiranofaciens* strains.

The assembled complete genome sizes of strains HL1 and M1 were 2,216,505 bp and 2,179,135 bp, respectively, with 37.5% of the same G+C contents. They comprised a circular chromosome of 2,156,113 bp and 2,180,483 bp, respectively, and a circular plasmid of 36,022 bp and 23,022 bp, respectively. For the HL1 genome, a total of 2225 predicted protein coding sequences (CDSs) were found, with 15 ribosomal RNAs (rRNAs) and 64 transfer RNAs (tRNAs). Meanwhile, the M1 genome had 2208 CDSs, 15 rRNAs, and 64 tRNAs (Figure 3A and Table 2). The general genomic features were almost the same in all six strains. The differences in genomic information may be a result of the genetic backgrounds of the different subspecies or strains. The interplay of sequencing quality, read length, sequencing depth and the assembler could also have affected the sequencing results [29]. It is worth noting that HL1 and M1 possessed seven clustered, regularly interspaced, short palindromic repeats (CRISPR), whereas DSM 10550^T^, KR, ATCC 43761^T^ and ZW3 had six, two, one and one, respectively. The CRISPR-Cas system cleaves phage and plasmid DNA, showing promise for self-defense [30]. Higher repeated CRISPR in HL1 and M1 than other strains suggested that CRISPR may play an important role in providing immunity against phages and plasmids.

The COG function of the gene showed that the top ten functions (Classes) of HL1 and M1 were as follows: replication, recombination and repair (Class-L); carbohydrate transport and metabolism (Class-G); transcription (Class-K); translation, ribosomal structure and biogenesis (Class-J); amino acid transport and metabolism (Class-E); inorganic ion transport and metabolism (Class-P); nucleotide transport and metabolism (Class-F); cell wall/membrane/envelope biogenesis (Class-M); and energy production and conversion (Class-C) (Table 3). These were similar to other *L. kefiranofaciens* strains [30], showing no difference between the two subspecies or the strains.

#### 3.2.2. Whole CDS Venn Diagrams

Figure 3B shows Venn diagrams and an Upset plot of the coding sequences of the six *L. kefiranofaciens* strains and four *L. kefiranofaciens* subsp. *kefirgranum* strains, respectively. The numbers of unique genes in HL1, M1, DSM 10550^T^, KR, ATCC 43761^T^ and ZW3 were 39 (1.7%), 17 (0.8%), 99 (4.7%), 132 (6.1%), 39 (1.6%) and 95 (3.9%), respectively. The HL1 genome shared 98.3% of the gene with M1. Comparing the HL1 and M1 genomes with the four *L. kefiranofaciens* subsp. *kefirgranum* strains, approximately 85% of the genes were orthologous. The unique genes could provide information related to the various properties and functionalities of the two subspecies and strains of *L. kefiranofaciens*.

### 3.3. Pan-Genome Analysis in HL1 and M1

#### 3.3.1. Polysaccharide Synthesis

*L*. *kefiranofaciens* is a polysaccharide kefiran-producing species which is responsible for the formation of the kefir grains matrix and the viscous property of kefir milk [31]. Thus, polysaccharide synthesis-related genes were analyzed.

##### The Cluster of Orthologous Groups Function of Genes in EPS Related Subsystems

The cluster of orthologous groups (COG) function of genes in the SEED subsystem was first analyzed; it showed that except for the sortase enzyme in the “Gram-positive cell wall components” subcategory, the gene numbers of HL1 and M1 in the “capsular and extracellular polysaccharides” subcategory, “no subcategory” and “Gram-positive cell wall components” were identical (Table 4 and Supplementary Table S3). Compare with other *L. kefiranofaciens* strains, differences in gene numbers were observed in the “capsular and extracellular polysaccharides” subcategory. Three *L. kefiranofaciens* subsp. *kefirgranum* strains, i.e., HL1, M1, and DSM 10550^T^, demonstrated similar gene numbers in the “capsular and extracellular polysaccharides” subcategory with the genes involved in the “dTDP-rhamnose synthesis” and “rhamnose-containing glycans” subsystems (Table 4). dTDP-rhamnose is an important precursor of cell wall polysaccharides and rhamnose-containing EPS [32]. Various lactic acid bacteria [33,34,35,36] possess rhamnose in their cell walls; this may serve as the primary binding site for certain bacteriophages [37]. HL1 and M1, with dTDP-rhamnose synthesis genes and rhamnose-containing glycans, verified our previous study, in which we determined that the M1 cell wall contained rhamnose (unpublished data). The finding regarding genetic COG functions not only suggested that rhamnose in the cell wall and kefiran were strain-dependent, but also provided a possible explanation for the previous CRISPR discovery. The higher repeated CRISPR in HL1 and M1 might be needed for self-defense against bacteriophage due to the presence of rhamnose in the cell wall.

##### Identification of the HL1 and M1EPS Biosynthetic Gene Cluster 

**Figure 4:**
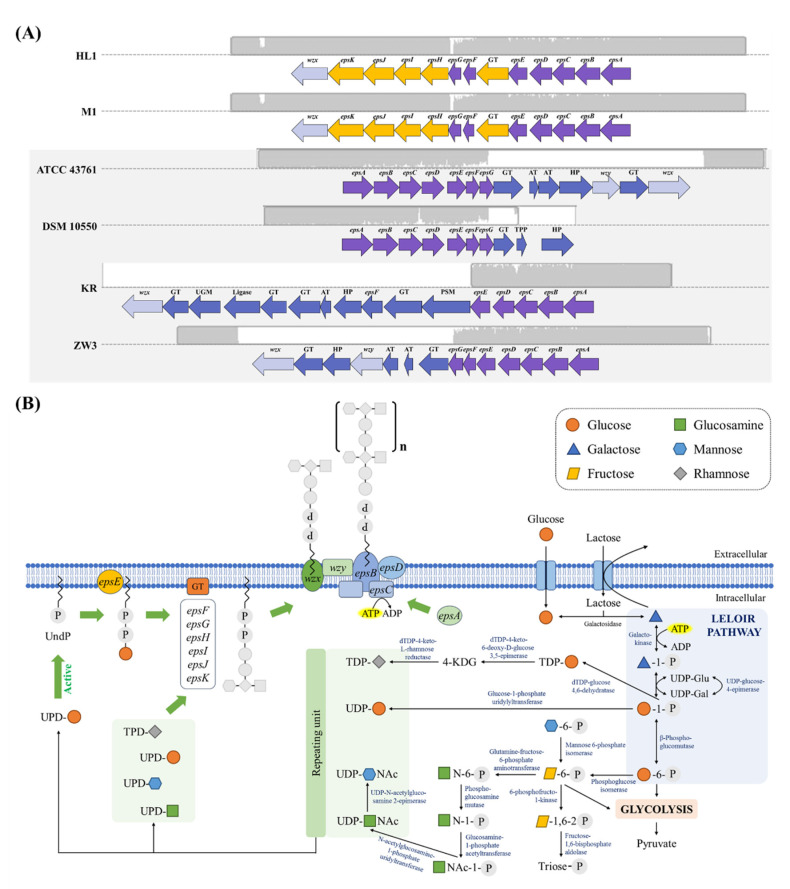
(**A**) Genetic organization of *eps* gene clusters in *L. kefiranofaciens* strains. Gene functional groups are marked with different colors. (**B**) Proposed biosynthesis pathway for the exopolysaccharide (EPS) production according to the Kyoto Encyclopedia of Genes and Genomes (KEGG) analysis.

A genomic comparison between the organization of EPS gene clusters in *L. kefiranofaciens* subsp. *kefirgranum* HL1 and M1, based on the putative or established functions of these products, is provided in Figure 4A. Four other *L. kefiranofaciens* strains (DSM 10550^T^, KR, ATCC 43761^T^, and ZW3) were used as references for the DNA sequences of the putative EPS gene clusters. The results indicated that HL1 and M1 possessed 13 genes (see Supplementary Table S4) which were located in the same orientation (Figure 4A). However, the EPS gene cluster in Wzy (polysaccharide polymerase) was different in HL1 and M1; this cluster encodes the functional protein related to the biosynthesis of repeating units. Wzy polysaccharide polymerase exhibits low sequence conservation in species with no Wzy homologues and with X-ray crystal structures [38]. Additionally, the protein encoded by *epsE* in strains HL1 and M1 demonstrated 94% identity with *Lactobacillus helveticus*. It was annotated as a priming glycosyltransferase (EC 2.7.8.6) which transfers the first sugar of each subunit of an EPS molecule. This enzyme plays an important role in EPS biosynthesis in Gram-positive lactic acid bacteria [39,40].

We also found three genes (*epsI, epsJ* and *epsK*) which were capable of encoding putative glycosyltransferases in the central portion of the putative EPS locus of HL1 and M1; there were considered to be distinct in the genomes, compared to those of other *L. kefiranofaciens* strains (see Figure 4A, Supplementary Table S4). An earlier study [40] revealed that the function of genes encoding glycosyltransferases in *Lactobacillus* was to transfer the monosaccharides of the EPS subunit in a sugar- and glycoside linkage-dependent manner. The three unique genes encoding glycosyltransferases in *L. kefiranofaciens* HL1 and M1 are probably responsible for the key enzymes producing unique EPS.

Based on our bioinformatic analysis, a biosynthetic model of EPS in *L. kefiranofaciens* HL1 and M1 is proposed (Figure 4B). The full biosynthetic process can be divided into two separate steps. The first involved the generation of activated sugar precursors from the metabolism of carbon in the cytoplasm. These enzymes, with the corresponding genes, indicated that *L. kefiranofaciens* HL1 and M1 possess multi-metabolic routes, including phosphoenolpyruvate, the sugar phosphotransferase system (PTS) and the Leloir pathway, which is involved in the generation of activated sugar precursors for EPS synthesis during the catabolism of glucose/lactose.

Among the aforementioned enzymes, fifteen were involved in UDP-glucose, UDP-galactose, UDP-mannose and TPD-glucosamine in the HL1 and M1 genomes. The number and type of monosaccharide nucleotides influence the composition and production of EPS [41,42]. This finding may also partially explain the differences in EPS yield and compositions between the strains of *L. kefiranofaciens* subsp. *kefirgranum* and those of *L. kefiranofaciens* subsp. *kefiranofaciens.* However, the gene encoded β-phospho-glucomutase (*β-PGM*) was not found in the Leloir pathway of the HL1 and M1 genomes. A previous study [43] which deleted β-phosphoglucomutase of *Lactococcus lactis* showed that the mutation did not influence growth, cell composition or product formation when glucose/lactose was used as the carbon source, but significantly reduced the maximum specific growth rates with maltose or trehalose as the carbon source. Thus, the lack of β-phospho-glucomutase in HL1 and M1 may affect the utilization of maltose/trehalose; this was consistent with the API 50 CHL result.

The second step (Figure 4B) was the Wzy pathway, connected to committed cell membrane-associated assembly and the polymerization of polysaccharides. *L. kefiranofaciens* M1 and HL1 possessed the following enzymes, characterized into three functional groups: (1) polysaccharide assembly function, including priming glycosyltransferase (*epsE*), flippase (*wzx*), polysaccharide polymerase (*wzy*) and phosphotransferase (*epsA*); (2) glycosyltransferase (*epsF*, *epsG*, *epsH*, *epsI*, *epsJ*, *epsK*); and (3) the phosphoregulatory system, including tyrosine kinase (*epsB, epsC*) and phosphotyrosine phosphatase (*epsD*), that regulate the polysaccharide assembly process. Both strains demonstrated a similar Wzy pathway to those of other *L. kefiranofaciens*, suggesting that this pathway was the conserved region in the *eps* genetic cluster. However, other than the conserved region, different regions of the *eps* genetic cluster in *Lactobacillus* could form EPSs with varied structures and molecular weights [44].

#### 3.3.2. Glycogen Metabolism and Stress Response

**Figure 5:**
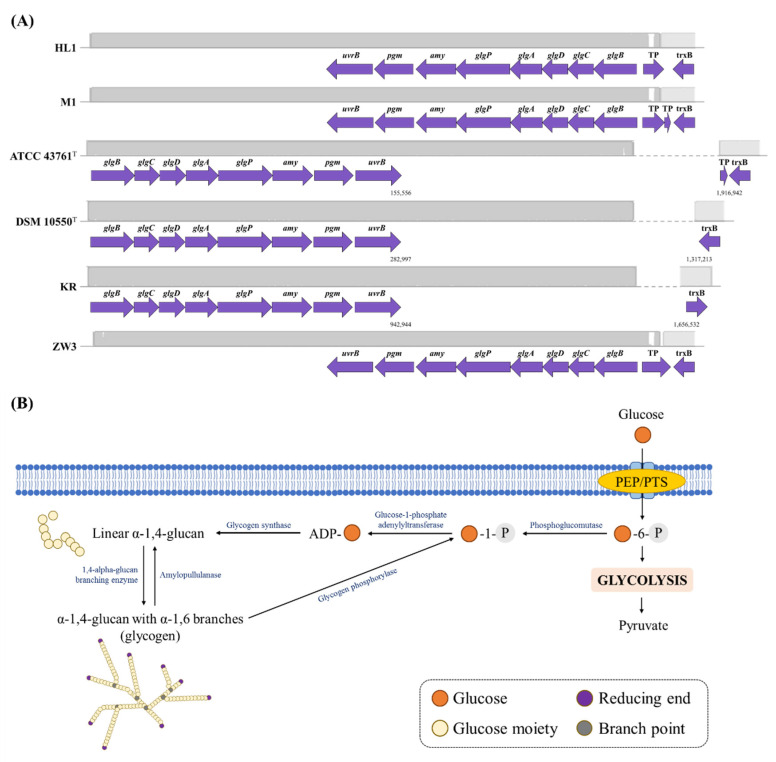
(**A**) Genetic organization of the glycogen metabolism gene clusters in *L. kefiranofaciens* strains. Gene functional groups are marked with different colors. (**B**) The proposed biosynthesis pathway in *L. kefiranofaciens* strains for glycogen production, according to the KEGG analysis.

A complete glycogen metabolism (*glg)* operon was found in the HL1 and M1 genome (see Figure 5, Supplementary Table S5). Both strains possessed the following glycogen biosynthetic enzymes encoded by the corresponding genes: phosphoglucomutase (*pgm*), glycogen phosphorylase (*glgP*), glycogen synthase (*glgA*), glycogen biosynthesis protein GlgD (*glgD*), glucose-1-phosphate adenylyltransferase (*glgC*) and 1,4-alpha-glucan branching enzyme GlgB (*glgB*), similar to the other four strains (Figure 5A). According to a bioinformatic analysis, a biosynthetic model of glycogen in *L. kefiranofaciens* HL1 and M1 is proposed (see Figure 5B). Glucose is phosphorylated into glucose-6-phosphate by transportation into the cytoplasm by the phosphoenolpyruvate:carbohydrate phospho-transferase system (PEP:PTS). Glucose-6-phosphate is then transformed into glucose-1-phosphate by phosphoglucomutase. Glucose-1-phosphate acts as a substrate for ADP-glucose synthesis, which is catalyzed by glucose-1-phosphate adenylyltransferase (GlgC) [45]. ADP-glucose is converted into linear α-1.4-glucose by glycogen synthase (GlgA) for the extending chain. The linear oligosaccharide is then converted into a highly branched structure by 1,4-alpha-glucan branching enzyme (GlgB). Two enzymes, i.e., glycogen phosphorylase (GlgP) and amylopullulanase (Amy), are involved in the catabolism of glycogen. GlgP catalyzes the sequential phosphorolysis of α-1,4-glucosyl linkages in the glucan chain from the non-reducing ends [46]. Finally, limited dextrins are generated by GlgP [47].

Complete *glg* operon, found in *L. kefiranofaciens* HL1 and M1, has also been detected in other *Lactobacillus* species, such as *L. acidophilus*, *L. amylovorus*, and *L. delbrueckii* subsp. *bulgaricus* [48]. Glycogen synthesis in bacteria is largely associated with the survival mechanism by storing carbohydrates and providing energy under diverse environments and stresses [48,49,50,51]. *L. kefiranofaciens* is the most abundant bacteria (around 90%) in kefir grains, playing a crucial role in early colonization, self-aggregation and grain formation [28,52]. Therefore, the complete *glg* operon in *L. kefiranofaciens* genomes suggests the importance of glycogen synthesis in this kefir species in terms of enabling colonization in kefir grains.

Additionally, one gene, HL1_0495 and M1_0491 in HL1 and M1 (NCBI Ref. KRL28325.1), respectively, coded the ImpB/MucB/SamB family protein, i.e., a a family of error-prone DNA polymerases involved in DNA repair [53]. The ImpB/MucB/SamB family of proteins has been reported to protect DNA from oxidative damage by directly binding to DNA [54]. The presence of the complete *glg* operon and the ImpB/MucB/SamB proteins in *L. kefiranofaciens* HL1 and M1 provide the potential for both strains to survive in harsh environments. This finding corresponds to our previous stress adaptation data [55]. The adaptation of *L. kefiranofaciens* M1 to heat, cold, acid and bile salts induced homologous tolerance and cross-protection against heterologous challenge through the increased synthesis of stress proteins.

#### 3.3.3. Cell Surface Adhesins

By the BLASTx analysis of the complete genomes of *L. kefiranofaciens* HL1 and M1, three mucus-binding proteins (NCBI Ref. AEG40448.1, KRL28865.1 and WP_013854242.1) and two LPXTG cell wall anchor domain-containing proteins (NCBI Ref. WP_126096172.1 and WP_054640578.1) were identified in each strain with high homology with mucus-binding domains, suggesting some functional similarities. LPXTG cell wall anchor domain-containing proteins have been reported to contain a C-terminal cell wall sorting signal with a sequence of amino acids; these proteins are connected to the cell wall by sortase A (*SrtA*) in lactic acid bacteria [56]. Other adhesion related genes, such as glycosylated streptococcal protein B (*GspB*), with affinity for sialic acid residues in mucins, and the mucus adhesion-promoting protein (*mapA*), were not found in HL1 and M1. In our previous study in germ free mice, *L. kefiranofaciens* M1 did not demonstrate a strong adhesion ability [57], probably due to the lack of certain adhesion-related genes.

### 3.4. The Unique Genes in HL1 and M1

A comparison of the full chromosome alignments of HL1 and M1 revealed a significant amount of genetic information about the two strains. The number of unique genes in *L. kefiranofaciens* HL1 and M1 were 72 and 32, respectively. The unique genes in HL1 comprised 52 hypothetical protein genes and 20 encoded genes (see Table 5, Supplementary Figure S5). The unique genes in M1 comprised 31 hypothetical protein genes and one encoded gene with the function of producing cold shock protein 2.

Adenine phosphoribosyltransferase, arginine deiminase, ornithine carbamoyltransferase and carbamate kinase 1, all of which were identified in HL1, are important enzymes in the arginine deiminase (ADI) pathway for arginine degradation. This pathway has been reported to contribute to ATP and ammonia production, resulting in enhanced viability under anaerobiosis with arginine induction in *Lactobacillus sakei* [58]. Additionally, arginine deiminase (*arcA* gene) has been considered as a potential anticancer agent [59] and an inhibitor of cell proliferation in various cancer cell lines [58,60]. The ability of *L. kefiranofaciens* to degrade arginine by the ADI pathway has never been described in the literature, and its physiological role remains unclear. The presence of the ADI pathway in HL1 suggests that this strain may increase stress tolerance under harsh environments, as well as providing certain health benefits.

Another unique gene, *cysK*, which is related to cysteine synthase, was identified in *L. kefiranofaciens* HL1. Cysteine could be a growth-limiting source in milk for microorganisms due to its low abundance in caseins [61,62]. Cysteine synthase might assist the survival of *L. kefiranofaciens* HL1 under low-cysteine-level conditions. However, cysteine and methionine are precursors of odor-active volatile sulfur compounds which are generated during fermentation [63]. The presence of this gene, which is involved in the metabolism of sulfur-containing cysteine in HL1, may suggest that this species could produce cysteine in milk and significantly influence flavor formation, especially in cheese ripening.

## 4. Conclusions

In the present study, we successfully classified *L. kefiranofaciens* into two subspecies, namely, *L. kefiranofaciens* subsp. *kefiranofaciens* and *L. kefiranofaciens* subsp. *kefirgranum*, based on phenotypic- and genotypic-based strain identification methods. Consequently, our strains, HL1 and M1, were identified as *L. kefiranofaciens* subsp. *kefirgranum*. Through a comparative whole genome sequence analysis, we then investigated the potential niche-specific genes and pathways among the two subspecies. The findings provided gene-level information with which to elucidate the differences in EPS composition and yield among the two subspecies and strains. We also provided the first report of the strain-specific ability of *L. kefiranofaciens* subsp. *kefirgranum* to degrade arginine via the ADI pathway; however, the physiological role of this remains unclear. The unique genes found in *L*. *kefiranofaciens* subsp. *kefirgranum* HL1 and M1 partially verified our previous findings of different functionalities. The novel findings on the potential genes and pathways of the two *L. kefiranofaciens* subspecies could be applied for further functionality predictions within the context of potential probiotic screening. This information could also be of use in elucidating their roles in kefir grains.

## Figures and Tables

**Table 1:** Differential carbohydrate fermentation characteristics of *L. kefiranofaciens* strains HL1 and M1, and their closest related type strains by API 50 CHL system. +, Positive; −, negative; w, weakly positive.

Carbon Source	HL1	M1	subsp. *kefirgranum* BCRC 80410^T^	subsp. *kefiranofaciens* BCRC 16059^T^
Amygdalin	−	−	+	−
Arbutin	−	−	+	−
Salicin	w	−	+	−
d-Cellobiose	−	−	+	−
d-Maltose	−	−	+	+
d-Melibiose	+	+	−	−
d-Sucrose	+	−	+	+
d-Trehalose	−	−	+	−
d-Raffinose	−	−	−	+
Gentiobiose	−	−	+	−
Aesculin	+	+	+	−

**Table 2:** General genomic features of *Lactobacillus kefiranofaciens* strains.

Subspecies	*kefirgranum*				*kefiranofaciens*	
Strain	HL1	M1	DSM 10550^T^	KR	ATCC 43761^T^	ZW3
Accession No.	GCA_023674385.1	GCA_023674405.1	GCA_001434195.1	GCA_002276565.1	GCA_900103655.1	GCA_000214785.1
Genome size (Mbp)	2.18	2.16	2.09	2.13	2.28	2.35
Contigs	2	2	138	97	84	3
GC content (%)	37.5	37.5	37.5	37.4	37.2	37.4
CDS number	2258	2208	2089	2141	2346	2423
CRISPR	7	7	6	2	1	1
rRNA	15	15	3	3	9	12

**Table 3:** Cluster of orthologous group categories (COGs) of *Lactobacillus kefiranofaciens* strains.

COG Class	Function	Gene Number					
		HL1	M1	DSM 10550^T^	KR	ATCC 43761^T^	ZW3
**L**	Replication, recombination and repair	252	235	176	183	215	258
**G**	Carbohydrate transport and metabolism	176	174	167	163	169	170
**K**	Transcription	164	163	171	156	165	164
**J**	Translation, ribosomal structure and biogenesis	151	151	154	154	154	154
**E**	Amino acid transport and metabolism	127	120	125	128	131	132
**P**	Inorganic ion transport and metabolism	120	119	120	118	125	125
**F**	Nucleotide transport and metabolism	109	108	107	113	114	114
**M**	Cell wall/membrane/envelope biogenesis	96	93	86	86	97	96
**C**	Energy production and conversion	66	66	66	74	71	71
**V**	Defense mechanisms	57	57	56	57	62	60
**I**	Lipid transport and metabolism	50	47	52	49	57	57
**H**	Coenzyme transport and metabolism	52	50	51	51	50	50
**U**	Intracellular trafficking, secretion, and vesicular transport	42	42	40	41	54	54
**O**	Posttranslational modification, protein turnover, chaperones	42	42	41	45	48	48
**T**	Signal transduction mechanisms	37	37	39	41	41	41
**D**	Cell cycle control, cell division, chromosome partitioning	35	35	35	34	36	35
**Q**	Secondary metabolites biosynthesis, transport and catabolism	10	10	10	10	13	13
**M**	Cell motility	7	7	8	9	7	7
**W**	Extracellular structures	5	5	4	5	10	10
**A**	RNA processing and modification	0	0	0	0	0	0
**B**	Chromatin structure and dynamics	0	0	0	0	0	0
**Y**	Nuclear structure	0	0	0	0	0	0
**Z**	Cytoskeleton	0	0	0	0	0	0
**S**	Function unknown	429	420	403	431	430	453
**Total**		**2252**	**2200**	**2115**	**2163**	**2287**	**2360**

**Table 4:** Comparison of genes related to the “Cell Wall and Capsule” SEED Category of *L. kefiranofaciens* strains.

Subcategory	HL1	M1	DSM 10550^T^	KR	ATCC 43761^T^	ZW3
**Capsular and extracellular polysaccharides**	**15**	**15**	**15**	**4**	**4**	**4**
dTDP-rhamnose synthesis	4	4	4	–	–	–
Exopolysaccharide biosynthesis	4	4	5	4	4	4
Rhamnose-containing glycans	7	7	6	–	–	–
**No subcategory**	**7**	**7**	**7**	**7**	**7**	**7**
Murein hydrolases	2	2	2	2	2	2
Recycling of peptidoglycan amino acids	1	1	1	1	1	1
UDP-*N*-acetylmuramate from fructose-6-phosphate biosynthesis	4	4	4	4	4	4
**Gram-Positive cell wall components**	**17**	**16**	**17**	**17**	**17**	**17**
d-Alanyl Lipoteichoic acid biosynthesis	3	3	3	3	3	3
Sortase	1	–	1	1	1	1
Teichoic and lipoteichoic acids biosynthesis	13	13	13	13	13	13
**Total**	**39**	**38**	**39**	**28**	**28**	**28**

**Table 5:** Unique genes in *L. kefiranofaciens* strain HL1, as compared with M1.

Locus_Tag	Start Position	End Position	ORF Length	% Identity	NCBI-Ref	Annotation
HL1_0556	539793	540485	693	99.86	WP_013853943.1	Class A sortase
HL1_0557	540610	542883	2274	99.74	WP_095341978.1	Single-stranded-DNA-specific exonuclease RecJ
HL1_0558	542989	543516	528	99.81	WP_013853945.1	Adenine phosphoribosyltransferase
HL1_0559	543772	546219	2448	99.80	KRL29238.1	Phosphoenolpyruvate synthase
HL1_0560	547467	546355	1113	99.91	WP_013853947.1	DUF871 domain-containing protein
HL1_0561	548483	547587	897	99.89	WP_013853948.1	Cysteine synthase A
HL1_0562	549284	548532	753	99.47	KRL29241.1	Homoserine *O*-succinyltransferase
HL1_0563	549994	549431	564	100.00	KRL29242.1	Cardiolipin synthetase
HL1_0564	550920	549991	930	99.68	KRL29243.1	Cardiolipin synthase
HL1_0565	551058	551549	492	99.8	WP_095341979.1	Lactocepin S-layer protein
HL1_0566	553917	551605	2313	99.87	WP_056941039.1	HAD-IC family P-type ATPase
HL1_0567	554068	555297	1230	99.76	WP_056941040.1	Arginine deiminase
HL1_0568	555311	556351	1041	99.90	WP_013853953.1	Ornithine carbamoyltransferase
HL1_0569	556335	557285	951	99.47	KRL29248.1	Carbamate kinase
HL1_0570	557299	557835	537	100.00	WP_013853955.1	Citrate lyase holo-[acyl-carrier protein] synthase
HL1_0571	557920	559083	1164	99.91	WP_013853956.1	GNAT family *N*-acetyltransferase
HL1_0573	559251	559865	615	100.00	AEG40162.1	Transcriptional regulator
HL1_0575	560614	561282	669	100.00	WP_013853960.1	Antibiotic biosynthesis monooxygenase
HL1_0577	563110	561755	1356	99.85	WP_013853962.1	Aspartate kinase
HL1_0578	564618	563128	1491	99.73	WP_056941043.1	Threonine synthase

## Data Availability

The datasets used and/or analyzed in the current study are available from the corresponding author on reasonable request.

## Supplementary Materials

The following supporting information can be downloaded at: https://www.mdpi.com/article/10.3390/microorganisms10081637/s1, Figure S1. The enterobacterial repetitive intergenic consensus (ERIC)-PCR and five randomly amplified polymorphic DNA (RAPD) profiles of *L. kefiranofaciens* subsp. *kefirgranum* strains HL1 and M1, and their reference strains. Figure S2. Genotypic characteristics of *L. kefiranofaciens* subsp. *kefirgranum* HL1 and M1. Phylogenetic tree based on 16S rRNA gene sequences. Figure S3. Genotypic characteristics of *L. kefiranofaciens* subsp. *kefirgranum* HL1 and M1. Phylogenetic tree based on housekeeping gene sequences [(A) *pheS* and (B) *rpoA*]. Figure S4. Phylogenomic tree based on whole genome sequences of *L. kefiranofaciens* strains and their closely related species. Figure S5. The unique genes in *L. kefiranofaciens* subsp. *kefirgranum* strain HL1 as comparing with strain M1 by MAUVE. Table S1. Oligonucleotide primer used in the PCR amplification. Table S2. Average nucleotide identity (ANI) values and digital DNA-DNA hybridization (dDDH) prediction values between HL1 and M1 with their reference strains. Table S3. Comparison of SEED subsystem features of *L. kefiranofaciens* strains. Table S4. Annotation of EPS genes of *L. kefiranofaciens* HL1 and M1 by RAST and NCBI. Table S5. Annotation results of the glycogen metabolism gene cluster by RAST and NCBI.
