# Supplementary material for: Subspecies Classification and Comparative Genomic Analysis of Lactobacillus kefiranofaciens HL1 and M1 for Potential Niche-Specific Genes and Pathways
Source: Microorganisms. 2022 Aug 12;10(8):1637. doi: 10.3390/microorganisms10081637 (PMC9415760; doi:10.3390/microorganisms10081637)
Supplement: Supplementary file 1 [file microorganisms-10-01637-s001.zip › microorganisms-1854887-supplementary.pdf]

Microorganisms Supplementary materials for:

Subspecies classification and comparative genomic analysis of *Lactobacillus kefiranofaciens* HL1 and M1 for potential niche-specific genes and pathways

Sheng-Yao Wang[1†], Yen-Po Chen[2†], Ren-Feng Huang[1], Yi-Lu Wu[1], Shang-Tse Ho[3], Kuan-Yi Li[1]
Koichi Watanabe[1*] and Ming-Ju Chen[1*]

[1] Department of Animal Science and Technology, National Taiwan University, Taipei 106037, Taiwan
[2] Department of Animal Science, National Chung Hsing University, Taichung 402204, Taiwan
[3] Department of Wood Based Materials and Design, National Chiayi University, Chiayi 600355, Taiwan

*Correspondence:
Ming-Ju Chen, P.h.D. (cmj@ntu.edu.tw)
Koichi Watanabe, P.h.D. (Koichi_wtnb@yahoo.co.jp)
Department of Animal Science and Technology, National Taiwan University
No. 50, Lane 155, Sec. 3. Keelung Rd., Taipei 106037, Taiwan

†These authors contributed equally to this work and share first authorship

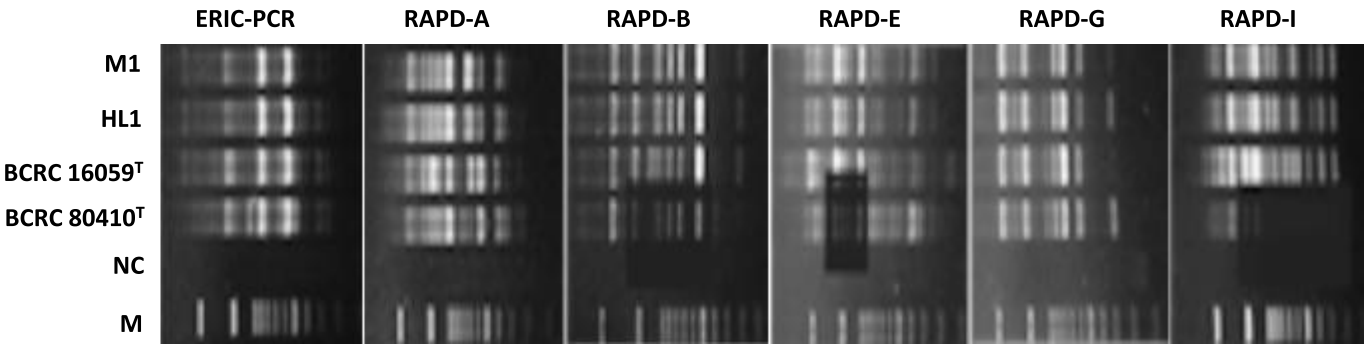

Figure S1. The enterobacterial repetitive intergenic consensus (ERIC)-PCR and five randomly amplified polymorphic DNA (RAPD) profiles of *L. kefiranofaciens* subsp. *kefirgranum* strains HL1 and M1, and their reference strains. Lanes M, 100 bp ladder DNA size marker; NC: negative control.

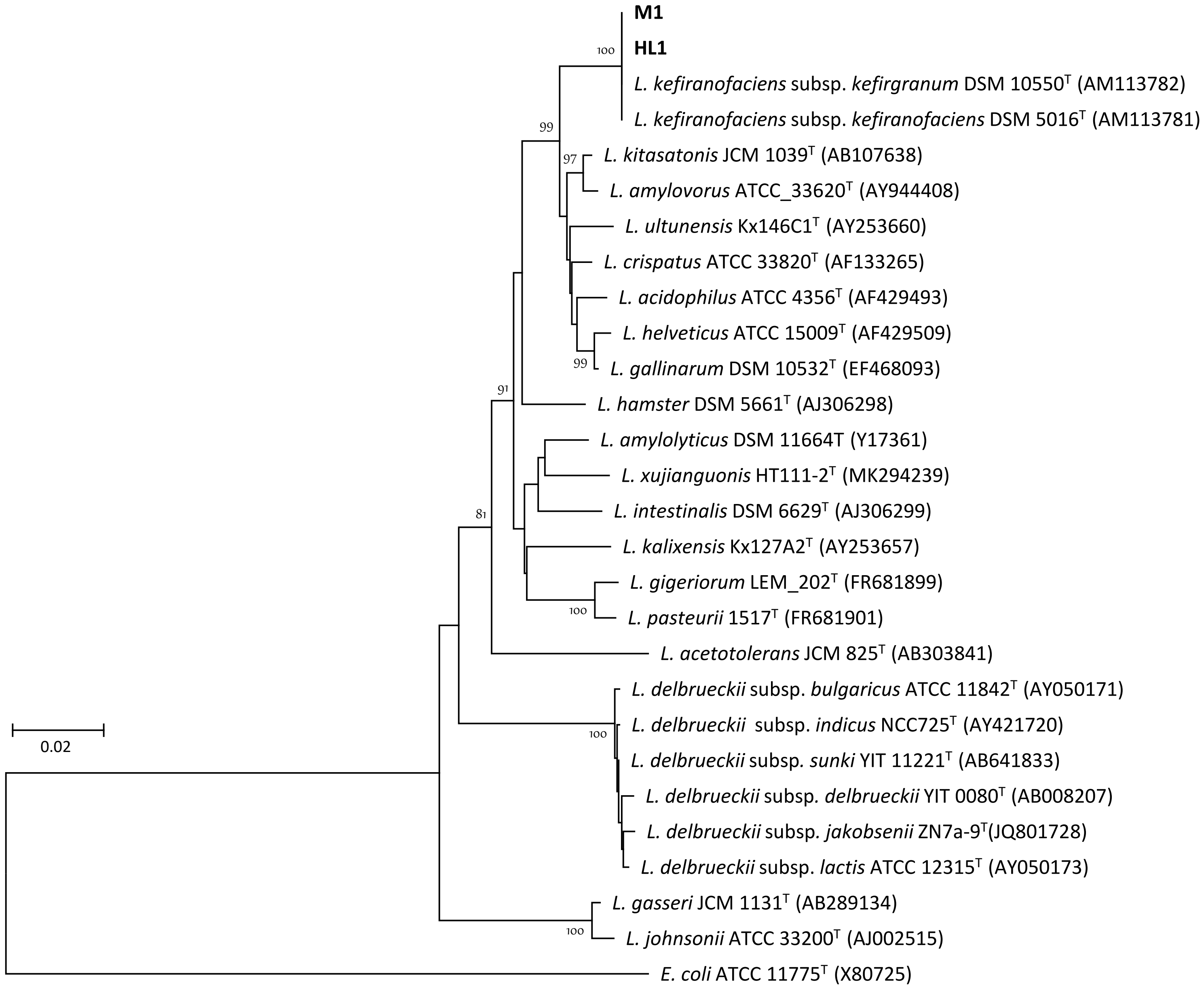

Figure S2. Genotypic characteristics of *L. kefiranofaciens* subsp. *kefirgranum* HL1 and M1. Phylogenetic tree based on 16S rRNA gene sequences. The tree was reconstructed by the neighbor-joining method with Kimura's two parameter model. Bootstrap values (>70%) with 1000 replications are give at nodes. Bar, 2% sequence divergence.

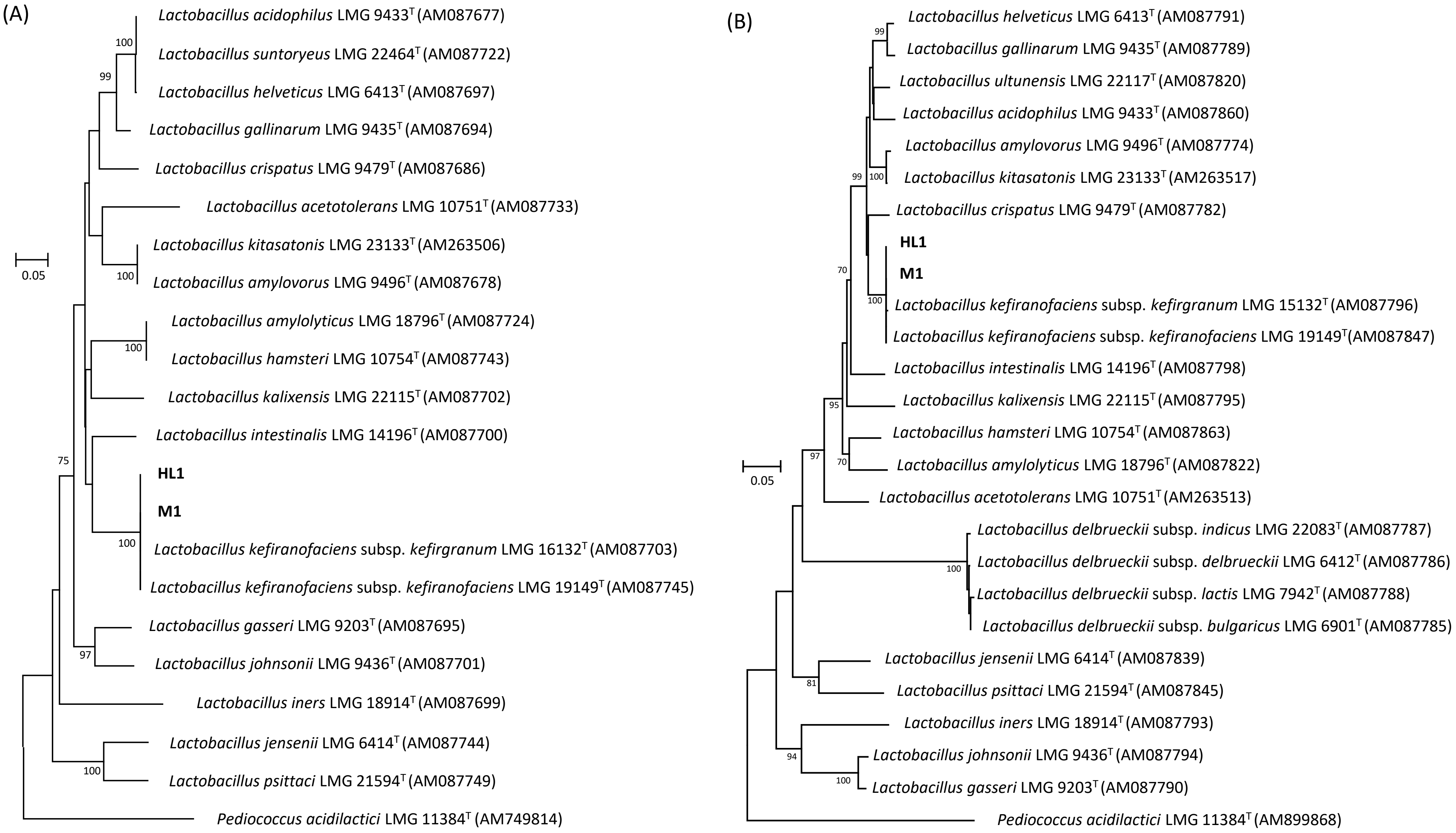

Figure S3. Genotypic characteristics of *L. kefiranofaciens* subsp. *kefirgranum* HL1 and M1. Phylogenetic tree based on housekeeping gene sequences [(A) *pheS* and (B) *rpoA*]. The trees were reconstructed by the neighbor-joining method with Kimura's two parameter model. Bootstrap values (>70%) with 1000 replications are give at nodes. Bars, 5% sequence divergence.

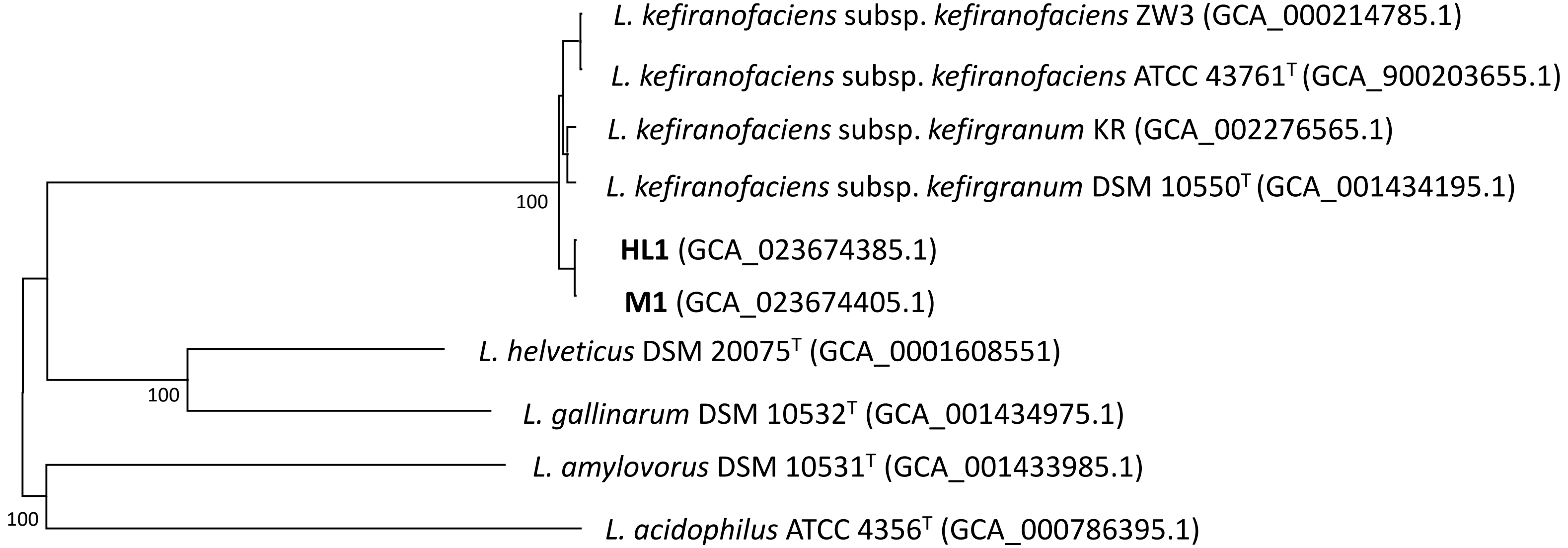

Figure S4. Phylogenomic tree based on whole genome sequences of *L. kefiranofaciens* strains and their closely related species. Tree inferred with FastME 2.1.6.1 Genomic BLAST Distant Phylogeny (GBDO) distances calculated from genome sequences. The branch lengths are scaled in terms of GBDP distance formula d5. The numbers above branches are GBDP pseudo-bootstrap support values from 100 replications, with an average branch support of 51.1%.

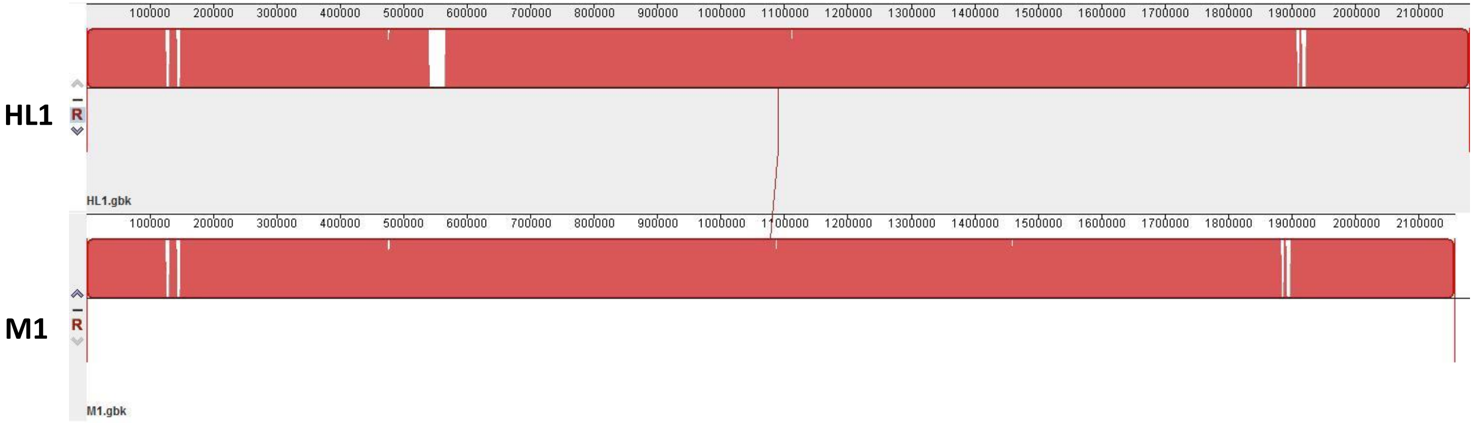

Figure S5. The unique genes in *L. kefiranofaciens* subsp. *kefirgranum* strain HL1 as comparing with strain M1 by MAUVE.

Table S1. Oligonucleotide primers used in the PCR amplification

Primer	Sequence (5'→3')	Target	Position	Reference
8F	AGAGTTTGATCMTGGCTCAG	16S rRNA gene	8–27	15
350F	GGGAGGCAGCAGTGGGGAATATTGC	16S rRNA gene	346–370	15
930F	GCACAAGCGGTGGAGCATGTGG	16S rRNA gene	933–954	15
520R	ACCGCGGCTGCTGGC	16S rRNA gene	804–787	15
15R	AAGGAGGTGATCCARCCGCA	16S rRNA gene	1541–1522	15
pheS 21F	CAYCCNGCHCGYGAYATGC	*pheS* gene	557–575	20, 21
pheS 23R	GCRTGRACCATVCCNGCHCCC	*pheS* gene	968–949	20, 21
rpoA 21F	ATGATYGARTTTGAAAAACC	*rpoA* gene	1–20	20, 21
rpoA 23R	ACHGTRTTRATDCCDGCRCG	*rpoA* gene	802–763	20, 21
ERIC 1	ATGTAAGCTCCTGGGGATTCAC	Genomic DNA		22
ERIC 2	AAGTAAGTGACTGGGGTGAGCG	Genomic DNA		22
RAPD-A	CCGCAGCCAA	Genomic DNA		23
RAPD-B	AACGCGCAAC	Genomic DNA		23
RAPD-E	GGCGTCGGTT	Genomic DNA		23
RAPD-G	CGAACTAGAC	Genomic DNA		23
RAPD-I	CTCAGGTCGC	Genomic DNA		23

Table S2. Average nucleotide identity (ANI) values and digital DNA-DNA hybridization (dDDH) prediction values between HL1 and M1 with their reference strains. The values on the upper right are dDDH values (%), and the values on the left are the ANI values (%)

	Species	Subspecies	strain	Accession No.	1	2	3	4	5	6	7	8	9	10
1	*L. kefiranofaciens*	*kefirgranum*	**HL1**	GCA_023674385.1	**100**	99.8	95.9	96.0	93.8	93.7	22.7	23.9	24.3	27.6
2			**M1**	GCA_023674405.1	99.98	**100**	95.9	96.2	93.8	93.8	22.7	23.9	24.4	27.8
3			DSM 10550^{T}	GCA_001434195.1	99.49	99.49	**100**	97.8	95.9	95.5	22.5	23.6	24.1	26.9
4			KR	GCA_002276565.1	99.46	99.50	99.72	**100**	95.8	95.4	22.5	23.6	24.1	27.5
5		*kefiranofaciens*	ATCC 43761^{T}	GCA_900103655.1	99.27	99.31	99.48	99.53	**100**	99.6	22.5	23.7	24.0	27.4
6			ZW3	GCA_000214785.1	99.27	99.31	99.52	99.50	99.96	**100**	22.6	23.9	24.1	27.6
7	*L. acidophilus*		ATCC 4356^{T}	GCA_000786395.1	79.32	79.29	79.53	79.31	79.29	79.29	**100**	24.6	24.1	24.3
8	*L. amylovorus*		DSM 20531^{T}	GCA_001433985.1	80.05	80.11	79.92	80.05	80.21	79.88	81.96	**100**	27.8	25.2
9	*L. gallinarum*		DSM 10532^{T}	GCA_001434975.1	79.41	79.41	79.48	79.60	79.42	79.50	79.63	79.98	**100**	38.9
10	*L. helveticus*		DSM 20075^{T}	GCA_000160855.1	83.02	83.03	82.47	83.20	82.97	82.88	80.85	81.27	80.23	**100**

Supplementary Table S3. Comparison of SEED subsystem features of *L. kefiranofaciens* strains. Genome sequences of L1, M1, DSM 10550^{T}, KR, ATCC 43761^{T}, and ZW3 were uploaded to the SEED Viewer server independently. Functional roles of RAST annotated genes were assigned and grouped in subsystem feature categories.

	subsp. *kefirgranum*				subsp. *kefiranofaciens*	
SEED subsystems	**HL1**	**M1**	DSM 10550^{T}	KR	ATCC 43761^{T}	ZW3
Protein Metabolism	122	122	111	109	117	123
Carbohydrates	116	115	106	108	131	130
Amino Acids and Derivatives	85	71	82	84	73	75
Nucleosides and Nucleotides	71	70	68	76	75	75
DNA Metabolism	68	68	66	65	65	64
Cofactors, Vitamins, Prosthetic Groups, Pigments	55	55	58	57	56	56
Cell Wall and Capsule	43	42	42	30	31	30
Fatty Acids, Lipids, and Isoprenoids	33	33	33	33	33	33
Virulence, Disease and Defense	32	32	33	45	31	32
RNA Metabolism	29	29	29	30	31	32
Membrane Transport	24	24	27	26	30	30
Regulation and Cell signaling	16	15	16	17	18	18
Respiration	15	15	15	15	20	19
Stress Response	8	8	8	8	11	11
Potassium metabolism	6	6	6	6	6	6
Dormancy and Sporulation	5	5	5	5	5	5
Iron acquisition and metabolism	4	0	4	4	5	5
Cell Division and Cell Cycle	4	4	4	4	4	4
Sulfur Metabolism	4	4	3	5	3	3
Miscellaneous	3	3	4	3	6	6
Metabolism of Aromatic Compounds	3	3	2	3	1	1
Secondary Metabolism	2	2	2	2	1	1
Motility and Chemotaxis	1	1	1	0	0	0
Phosphorus Metabolism	1	1	0	0	0	0
Photosynthesis	0	0	0	0	0	0
Phages, Prophages, Transposable elements, Plasmids	0	0	0	3	0	0
Nitrogen Metabolism	0	0	0	0	0	0
Total	750	728	725	738	753	759

Table S4. Annotation results of EPS cluster by RAST and NCBI

Gene	Annotation	
	RAST	NCBI
epsA	Cell envelope-associated transcriptional attenuator	LCP family protein
epsB	Tyrosine-protein kinase transmembrane modulator	Exopolysaccharide biosynthesis protein
epsC	Tyrosine-protein kinase	Tyrosine-protein kinase
epsD	Manganese-dependent protein-tyrosine phosphatase	Exopolysaccharide biosynthesis protein
epsE	Undecaprenyl-phosphate galactosephosphotransferase	Sugar transferase
epsF	Polysaccharide biosynthesis protein	UDP-N-acetylglucosamine--LPS N-acetylglucosamine transferase
epsG	Glycosyltransferase, family 28	multidrug MFS transporter
epsH	hypothetical protein	TPA: capsular polysaccharide synthesis protein
epsI	Glycosyltransferase, family 28	Glycosyltransferase
epsJ	hypothetical protein	Capsular polysaccharide synthesis protein
epsK	Alpha-L-Rha-alpha-1,3-L-rhamnosyltransferase	Glycosyltransferase family 2 protein

Table S5. Annotation results of the glycogen metabolism gene cluster by RAST and NCBI

Gene	NCBI-Ref	Annotation
uvrA	WP_056941077.1	excinuclease ABC subunit UvrA
uvrB	WP_013854444.1	excinuclease ABC subunit UvrB
pgm	WP_013854445.1	phospho-sugar mutase
amy	WP_056941076.1	glycoside hydrolase family 13 protein
glgP	WP_054640146.1	glycogen/starch/alpha-glucan phosphorylase
glgA	WP_054640145.1	glycogen synthase GlgA
glgD	KRL28923.1	glucose-1-phosphate adenylyltransferase
glgC	WP_013854450.1	glucose-1-phosphate adenylyltransferase
glgB	WP_056941074.1	1,4-alpha-glucan branching protein GlgB
TP	WP_095342487.1	ISL3 family transposase
trxB	WP_013854453.1	thioredoxin-disulfide reductase
